# The insulin receptor family in the heart: new light on old insights

**DOI:** 10.1042/BSR20221212

**Published:** 2022-07-18

**Authors:** Angela Clerk, Peter H. Sugden

**Affiliations:** School of Biological Sciences, University of Reading, Reading, U.K.

**Keywords:** insulin receptors, insulin signalling, intracellular signaling, regulation of metabolism

## Abstract

Insulin was discovered over 100 years ago. Whilst the first half century defined many of the physiological effects of insulin, the second emphasised the mechanisms by which it elicits these effects, implicating a vast array of G proteins and their regulators, lipid and protein kinases and counteracting phosphatases, and more. Potential growth-promoting and protective effects of insulin on the heart emerged from studies of carbohydrate metabolism in the 1960s, but the insulin receptors (and the related receptor for insulin-like growth factors 1 and 2) were not defined until the 1980s. A related third receptor, the insulin receptor-related receptor remained an orphan receptor for many years until it was identified as an alkali-sensor. The mechanisms by which these receptors and the plethora of downstream signalling molecules confer cardioprotection remain elusive. Here, we review important aspects of the effects of the three insulin receptor family members in the heart. Metabolic studies are set in the context of what is now known of insulin receptor family signalling and the role of protein kinase B (PKB or Akt), and the relationship between this and cardiomyocyte survival versus death is discussed. PKB/Akt phosphorylates numerous substrates with potential for cardioprotection in the contractile cardiomyocytes and cardiac non-myocytes. Our overall conclusion is that the effects of insulin on glucose metabolism that were initially identified remain highly pertinent in managing cardiomyocyte energetics and preservation of function. This alone provides a high level of cardioprotection in the face of pathophysiological stressors such as ischaemia and myocardial infarction.

The heart is a sophisticated pump essential for delivery of oxygenated blood and nutrients to the body. It relies on contractile cardiomyocytes, acting in synchrony, to pump blood around the body but, in adult mammals, there is negligible capacity for regeneration or replacement of these cells. Pathophysiological stresses imposed on cardiomyocytes can compromise their contractile ability or cause them to die. This reduces the ability of the heart to function properly and can result in heart failure, an increasing problem in society. Urgent remedies are needed to prevent cardiomyocyte dysfunction and death. Insulin has potent cardioprotective effects, particularly in the context of ischaemia with or without reperfusion, as occurs with myocardial infarction. Insulin acts via its cognate receptor, but there are two related receptors that activate similar signalling pathways. Here, we first provide an overview of the context of cardiac hypertrophy and heart failure, outlining the response of the heart to pathophysiological stresses. We then discuss the effects of insulin and the insulin family of receptors, describing key signalling elements and how these may affect cell survival. Finally, we discuss how insulin receptor family signalling may elicit cardioprotection.

## Cardiomyocyte growth and cardiac hypertrophy

Architecturally, the heart is fully formed at birth, but continues to grow through postnatal life, matching the increase in body size through to adulthood. In the embryo/foetus, cardiomyocytes proliferate to generate sufficient cells for the heart to form, but they become terminally differentiated in the perinatal/postnatal period [1,2]. Subsequent maturational growth of the heart to the adult size requires an increase in size and myofibrillar content of individual cardiomyocytes (maturational growth or eutrophy). The adult heart experiences numerous pathophysiological stresses that may demand an increase in cardiomyocyte contractile function. This is accommodated by cardiomyocyte hypertrophy (i.e. an increase in cell size) with associated cardiac hypertrophy (enlargement of the heart) [3]. Physiological hypertrophy (e.g. as occurs in pregnancy or with endurance exercise) is clearly beneficial, necessary and largely reversible on cessation of the stress [4]. Pathological hypertrophy may develop in diseases such as hypertension which increase cardiac workload, or following myocardial infarction where loss of blood supply to part of the myocardium causes cardiomyocytes to die [5,6]. The resulting deposition of inelastic fibrotic scar tissue increases the workload of surviving cardiomyocytes and these hypertrophy. Such “compensated” cardiac hypertrophy initially facilitates survival, but may decompensate, leading to heart failure. Decompensated hypertrophy is associated with further cardiomyocyte death, with loss of capillaries, inflammation and fibrosis.

Cardiomyocyte growth, whether maturational, physiological or pathological is an anabolic process and, although associated with changes in gene expression, it requires an underpinning increase in protein accretion [7]. Insulin and insulin-like growth factors (IGFs) act through related receptor tyrosine kinases (RTKs), namely insulin receptors (INSRs) and Type 1 IGF1 receptors (IGF1Rs) [8]. As we describe below, both promote protein accumulation in the heart and are implicated in cytoprotection. The role of the third related receptor, the insulin receptor-related receptor (INSRR), identified in 1989 [9], is only now being revealed.

## Regulation of cardiac metabolism by insulin and IGFs: impact on protein synthesis

One of the earliest effects of insulin to be established (and possibly the best characterised) was the stimulation of glucose uptake by insulin-sensitive tissues such as the heart [10]. In addition, insulin has a range of other effects on carbohydrate, lipid and protein metabolism: it increases the rate of glycolysis whilst promoting glycogen accumulation, it reduces the rate of lipolysis promoting fatty acid synthesis and decreasing fatty acid oxidation, it increases the rate of protein synthesis and it decreases the rate of protein degradation [11]. In the heart, there has been a focus on the effects of insulin to increase protein synthesis because it is a key feature of cardiac hypertrophy [7]. Thus, protein synthesis (measured by incorporation of the non-metabolised amino-acid phenylalanine) in ribosomes from rat hearts is stimulated by insulin and inhibited by diabetes [12], and cardiac protein synthesis is increased by perfusion of rat hearts with insulin (see, for example, [13,14]). Conversely, cardiac protein synthesis is inhibited by hypoinsulinaemia (diabetes, starvation) both *in vitro* and *in vivo* with loss of cardiac protein mass (see, for example, [14–17]). Analogous results are obtained in perfused hearts or isolated cardiomyocytes treated with IGF1 or IGF2 [18]. Insulin, IGF1 or IGF2 each increase the rate of protein synthesis to the maximimum limit of up to ∼80%, and the point of action is largely at the level of translational initiation [19]. In concert with its effects to increase the rate of protein synthesis, insulin inhibits global protein degradation [20,21], although the point of action remains ill-defined. Interestingly, degradation of the myofibrillar protein actin is increased by starvation in a manner which is unaffected by insulin [22]. This contrasts with phenylalanine release in the presence of cycloheximide (to inhibit protein synthesis) as a measure of global protein degradation, suggesting that insulin is selective in its effect on protein degradation. In conclusion, in the heart (as in other insulin-responsive tissues), insulin and IGFs act as ‘anabolic’ hormones.

Stimuli other than insulin increase the rate of protein synthesis in the heart and are overtly pro-hypertrophic causing an increase in cardiomyocyte size. These include α_1_-adrenergic receptor (α_1_-AR) agonists, but maximal stimulation of protein synthesis by, for example, the synthetic agonist phenylephrine, is less than that obtained by insulin [23]. Indeed, the only intervention capable of increasing cardiac protein synthesis to a similar degree as insulin or IGFs has so far proved to be exposure to pH_o_ above the ‘physiological’ value of 7.4, as shown with perfused rat hearts [24]. In contrast with insulin (which does not affect cardiac ‘contractility’), increased pH_o_ is positively inotropic, suggesting some mechanistic differences. This positive inotropic effect of increased pH_o_ could involve an effect on pH_i_ (pH_i_ is linearly coupled to pH_o_ with ΔpH_i_/ΔpH_o_ ∼ 0.4–0.5), since increased pHi (by increasing the ionisation of the acidic amino acid Ca^2+^-binding residues) increases Ca^2+^-binding to troponin C, a key regulator of contraction. These studies were conducted in the 1980s prior to the identification of any of the downstream signalling events associated with insulin receptor signalling. The hypothesis at the time was that understanding the mechanism of action of the effect of increased pH_o_ on cardiac protein synthesis may assist in understanding the signalling pathways utilised by the insulin receptor, but it took over 20 years to revisit this question.

## Insulin and IGF signalling in the heart

### INSRs and IGF1Rs: receptors for insulin, IGF1 and IGF2

**Figure 1:**
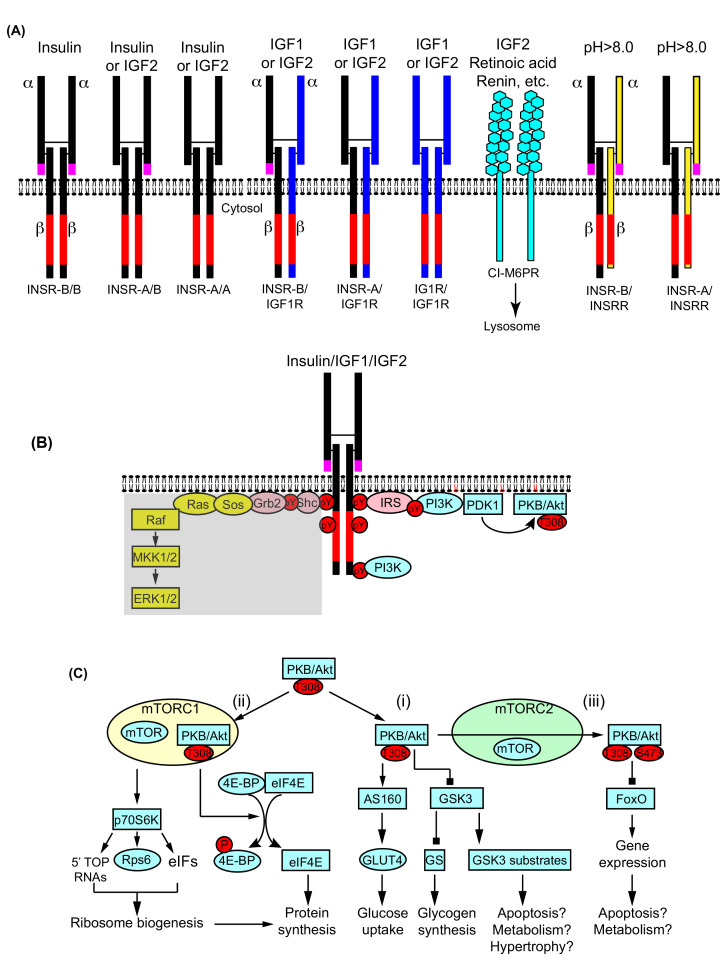
Insulin receptor family signalling (**A**) Insulin receptors (INSRs, black) and the related receptor family members, IGF1 receptors (IGF1Rs, blue) and insulin receptor-related receptors (INSRRs, yellow) are expressed at the cell surface as preformed heterotetramers, each comprising two hemi-receptors composed of one α- and one β-subunit. Each α- and β-subunit and the two α-subunits are cross-linked by disulphide bridges. The extracellular α subunits bind insulin or IGFs as indicated or are responsive to alkaline pH_o_. The β-subunits have a tyrosine kinase domain (red). Alternative splicing generates two isoforms of INSR, INSR-A and INSR-B, without or with (respectively) exon 11 (pink). Hybrid receptors can form between the family members. IGF2 also binds to the cation-independent mannose 6-phosphate receptor (CI-M6PR) that directs IGF2 and other molecules to the lysosome for degradation. (**B**) Insulin or IGFs bind to INSRs/IGF1Rs causing receptor phosphorylation and full activation of the tyrosine kinase, with additional phosphorylation of tyrosine residues (pY) on the β-subunit. These recruit adapter proteins including insulin receptor substrates (IRS) that become phosphorylated. IRS proteins recruit phosphoinositide 3′ kinase (PI3K) that stimulates production of phosphatidylinositol 3,4,5 *tris* phosphate in the membrane (red lipids), recruiting PDK1 and protein kinase B (PKB, also known as Akt). PDK1 phosphorylates PKB/Akt on Thr308. In some cells, INSRs/IGF1Rs also signal via Grb2 and the exchange factor Sos to the small G protein Ras. Activated Ras recruits Raf kinases that phosphorylate and activate mitogen-activated protein kinase kinases 1/2 (MKK1/2) that phosphorylate extracellular signal-regulated kinases 1 and 2 (ERK1/2). However, this pathway is not thought to be activated significantly in the heart and is shaded. (**C**) (i) PKB/Akt(T308) phosphorylates AS160 (Tbc1d4) to increase translocation of the glucose transporter GLUT4 to the membrane for glucose uptake. Phosphorylation and inhibition of glycogen synthase kinase 3 (GSK3) results in activation of glycogen synthase (GS) to increase glycogen synthesis. Other GSK3 substrates influence apoptosis, metabolism and cardiac hypertrophy. (ii) PKB/Akt(T308) activates mammalian target of rapamycin (mTOR) in mTOR complex 1 (mTORC1). Signalling via p70 ribosomal S6 kinase (p70S6K) increases ribosome biogenesis by increasing translation of RNAs with 5′ terminal oligopyrimidine (TOP) sequences), phosphorylation of Rps6 and regulation of other eukaryotic initiation factors (eIFs). mTORC1-dependent phosphorylation of 4E-BP causes it to dissociate from eIF4E that is then available to bind to the 5′ cap structure of mRNAs to increase translation and enhance protein synthesis. (iii) PKB/Akt(T308) is phosphorylated on Ser473 in mTORC2. Dually phosphorylated PKB/Akt(T308/S473) has greater activity and additional target specificity for forkhead transcription factors of the FoxO family. Phosphorylation of FoxO transcription factors inhibits their effects on gene expression, to influence apoptosis and metabolism.

The general structure of insulin receptor (INSR) was established in 1980 [25,26], with subsequent studies of this and the related Type 1 IGF1 receptor (IGF1R) providing insight into how insulin and IGFs bind to these receptors [27,28]. Important progress has been made in recent years in elucidating the atomic detail of the insulin and IGF1 receptors, along with ligand interactions as reviewed in, for example, [29,30]. Since the molecular detail of the receptors or receptor–ligand interactions is not the focus of this review, we will only present an overview of receptor structure and, to avoid confusion, we will use Online Mendelian Inheritance in Man (OMIM®) terminology for the genes/proteins as far as possible. INSRs and IGF1Rs are heterotetrameric receptors, preformed prior to insertion in the plasma membrane (Figure 1A). Each half of the receptor (hemi-receptor) is derived from a single polypeptide. Proteolytic cleavage of an internal cleavage site generates α- and β-subunits held together by a disulphide bridge. Additional proteolysis removes the N-terminal signal peptide. The two extracellular α-subunits are also linked via disulphide bridges to form a stable heterotetrameric α_2_β_2_ structure. The β-subunits each contain a single transmembrane domain and an intracellular protein tyrosine kinase. Insulin or IGFs interact with the α-subunits of INSRs or IGF1Rs, causing a conformational change that is transmitted through the transmembrane β-subunits to activate the innate protein tyrosine kinase. The β-chains transphosphorylate Tyr residues in the intracellular kinase domains to activate the kinase fully. Further phosphorylation of additional Tyr residues occurs, and other proteins are recruited to these sites, binding through SH2 or PTB domains (that recognise phospho-Tyr residues with C-terminal or N-terminal specificity determinants, respectively) [31]. Some are adapter proteins that become Tyr-phosphorylated by the receptor. This allows recruitment of further proteins to form a signalling complex or ‘signalosome’.

INSR and IGF1R have high sequence homology in the cytoplasmic tyrosine kinase domain, with greater divergence in the extracellular ligand-binding α-subunits [27,28]. Insulin and IGF1 can each bind to INSRs and IGF1Rs but, at physiological concentrations of circulating hormone, each receptor should only bind its cognate ligand. The mode of ligand binding to each receptor differs. A single molecule of insulin engages with both α-subunits of the INSR heterotetramer, and negative co-operativity between the hemi-receptors appears to reduce affinity for a second molecule [32]. In contrast, there is no co-operativity between IGF1R hemi-receptors, and IGF1R heterotetramers may bind one or two molecules of IGF1 [33,34]. This is important to consider since INSRs and IGF1Rs form hybrid receptors (Figure 1A), potentially a consequence of mass action during the process of receptor assembly [35–37]. These INSR/IGF1R hybrid receptors have similar affinity for IGF1 as IGF1R heterotetramers, and low affinity for insulin [38]. Since the hybrid receptors operate largely as IGF1Rs with respect to ligand binding, this increases the number of available receptors for IGF1 in the heart by almost 2-fold, most likely increasing sensitivity. A question remains as to whether signalling from INSR/IGF1R hybrid receptors is similar to IGF1R heterotetramers, given that downstream engagement of signalling intermediates to INSRs and IGF1Rs may differ, along with activation of protein kinases and effects on cell function [39,40]. Notably, approximately 90% of IGF1Rs in the heart form hybrid receptors with INSRs [41].

IGF2 is often considered to bind simply to the IGF2 receptor (IGF2R) [42]. This is the cation-independent mannose 6-phosphate receptor (CI-M6PR), a large glycoprotein that serves as a clearance receptor for IGF2 and other molecules, targeting them to the lysosome for degradation [43]. However, IGF2 binds with approximately equal affinity as IGF1 to IGF1Rs [44]. Apart from this, there are two isoforms of the INSR (INSR-A and INSR-B) generated by alternative splicing [45]. INSR-B contains an additional 12 residues derived from exon 11, towards the C-terminus of the α subunit and these receptors have high affinity for insulin whilst INSR-A receptors (lacking exon 11) have high affinity for IGF2 [46]. The ratio of INSR-A:INSR-B isoforms in the heart is approximately 1:2 in young, healthy rat hearts, but the proportion of INSR-A isoforms is higher in older animals and in diabetes [47]. Overall, there is potential for adult cardiac cells to respond to IGF2 via INSR-A receptors, IGF1Rs or hybrid INSR/IGF1Rs (Figure 1A). This potentially accounts for the similar effects of IGF1 and IGF2 on protein synthesis in the heart [18]. IGF2 is often considered as a developmental growth factor, but circulating levels of both IGF1 and IGF2 remain high in adults (IGF1: 100–300 ng/ml; IGF2: 400–1100 ng/ml [48,49]). However, the majority of both growth factors is associated with IGF binding proteins (IGFBPs) [50]. Free, unbound growth factors are unstable, having a short half-life (∼10 min) and circulating at low levels of 1–2 ng/ml [49]. Release of IGFs from IGFBPs requires proteolysis of the binding proteins [51]. This presumably occurs within target tissues and the reaction could be increased in pathological states. Both IGF1 and IGF2 could also be produced locally in the heart under pathological conditions and, since IGF1 or IGF2 can bind to INSRs with low affinity, a question remains about whether local production of these growth factors may be sufficiently high to trigger INSRs in addition to IGF1Rs. Despite the abundance of both IGF2 and its active receptors, the role of IGF2 in the heart has been generally ignored relative to IGF1 or insulin.

As mentioned above, INSRs and IGF1Rs become Tyr-phosphorylated on the intracellular β-subunits following activation, recruiting other proteins to the complex. Two key proteins in the insulin signalling system are insulin receptor substrates (IRS), IRS1 and IRS2, large adapter proteins which bind to the receptors via PTB domains [52]. All potential phosphorylation sites and the protein interactome for each of the insulin receptor family β-subunits, IRS1 and IRS2 have been mapped [39]. The precise sites which are used and the proteins that interact presumably depend on protein expression profiles in specific tissues or cell types, in addition to considerations of subcellular localisation and compartmentalisation. INSRs and IGF1Rs signal through IRS1/IRS2 in the heart (as in other tissues) [53], extending the potential cardiac interactome of these receptors. Despite the high homology between the INSR and IGF1R β-subunits, insulin, IGF1 and IGF2 have different effects, both on the intracellular signalling pathways they activate and downstream consequences for the cell [39,40].

### Protein kinase signalling from INSRs and IGF1Rs

A full summary the intracellular signalling pathways activated by INSRs and IGF1Rs is beyond the scope of this overview, and readers are referred to other reviews for full details [54–56]. Here, we aim to provide an outline of the signalling with reference to the knowledge of the events in the heart. Two key signalling pathways are potentially activated by the insulin receptor family (Figure 1B): phosphoinositide 3′ kinase (PI3K) signalling through protein kinase B (PKB, also known as Akt); and the original mitogen-activated protein kinases (MAPKs), the extracellular signal-regulated kinases 1/2 (ERK1/2).

### Signalling through PI3K and PKB/Akt

#### Activation of PKB/Akt

Of the proteins that bind to the insulin family receptors and IRS1/2, potentially the most prominent are the Type 1 PI3Ks [57,58]. INSRs and IGF1Rs have a direct docking site for PI3K at the C-terminus of the β-subunit (phospho-Y1351/1352), and also recruit PI3Ks via IRS1/2 [39]. PI3K phosphorylates phosphatidylinositol 4,5 *bis*phosphate (PIP_2_) in the membrane to produce phosphatidylinositol 3,4,5 *tris*phosphate (PIP_3_). This recruits the Ser/Thr protein kinases, phosphoinositide-dependent kinase 1 (PDK1) and PKB/Akt, allowing PDK1 to phosphorylate PKB/Akt on Thr308 to activate the kinase. PKB/Akt activates mammalian target of rapamycin (mTOR) within mTOR complex 1 (mTORC1). mTOR also operates in a separate complex, mTORC2, and promotes phosphorylation of PKB/Akt on Ser473. This increases PKB/Akt activity and extends the range of potential substrates [59]. As expected, insulin and IGF1 potently activate PKB/Akt in cardiomyocytes and the heart, with increased phosphorylation of both Thr308 and Ser473 [60–62], indicative of activation of mTORC1 and mTORC2 complexes. PKB/Akt serves as a hub for insulin family receptor signalling in the heart, as in other tissues, orchestrating a co-ordinated anabolic response to insulin associated with cell survival (Figure 1C) [55].

#### PKB/Akt substrates associated with metabolism and cell survival

Probably the best characterised direct substrates of PKB/Akt are glycogen synthase kinase 3 α and β (GSK3α/β) that are inhibited by PKB/Akt-mediated phosphorylation of Ser21 and Ser9, respectively. GSK3α/β phosphorylate various substrates including glycogen synthase, phosphorylation of which by GSK3α/β is inhibitory [63], the net effect of PKB/Akt being to increase glycogen synthesis (by inhibition of inhibition). Phosphorylation of GSK3α/β in response to insulin or IGF1 in the heart and cardiomyocytes is well-documented, although the emphasis is generally on its role in cardiac hypertrophy, rather than regulation of glycogen synthesis [64–66]. Overall, GSK3α/β activity prevents cardiac and cardiomyocyte hypertrophy, acting through a variety of potential mechanisms. These include phosphorylation and inhibition of several transcription factors, prevention of opening of the mitochondrial permeability transition pore (mPTP) and apoptosis and/or phosphorylation and degradation of β-catenin.

**Figure 2:**
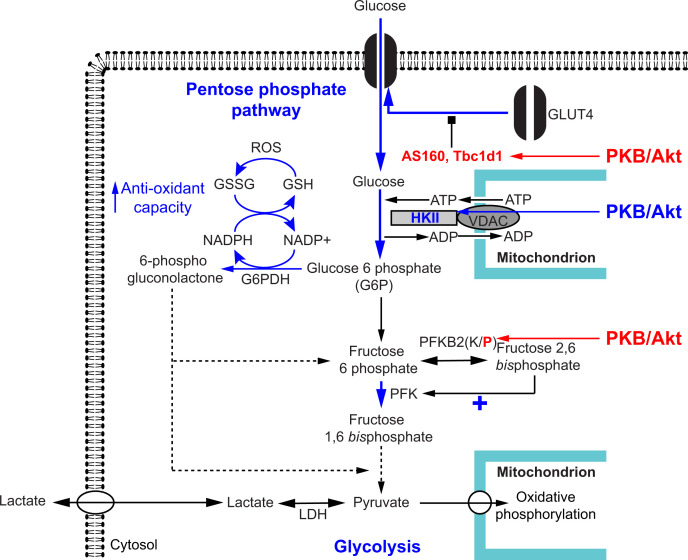
Glucose metabolism through the glycolytic and pentose phosphate pathways: regulation by PKB/Akt PKB/Akt phosphorylates RabGAPs (AS160/Tbc1d4, Tbc1d1) to prevent their inhibitory action (red type), increasing translocation of vesicles containing the glucose transporter GLUT4 to the membrane (blue arrow). This increases glucose uptake into the cell. Hexokinase II (HKII) converts glucose to glucose 6-phosphate (G6P). It is tethered to the mitochondrial membrane, interacting with the voltage-dependent anion channel (VDAC), allowing it to access ATP directly from the mitochondria, and facilitating direct transfer of ADP back into the mitochondria. HKII expression and activity is increased by PKB/Akt signalling (blue type) increasing production of G6P. Glycolysis (centre). G6P is converted to fructose 6-phosphate which is then converted to fructose 1,6-bisphosphate by phosphofructokinase (PFK) in the rate-limiting step of the pathway. Fructose 6-phosphate interconversion with fructose 2,6-*bis*phosphate is performed by the cardiac isoform of phosphofructokinase 2 (PFKB2), an enzyme with dual kinase (K) and phosphatase (P) activity. PKB/Akt phosphorylates and inhibits the phosphatase activity, resulting in accumulation of fructose 2,6-bisphosphate, an allosteric activator of PFK. This increases glycolytic flux to produce pyruvate for oxidative phosphorylation in the mitochondria. If oxidative phosphorylation is insufficient to manage additional pyruvate, pyruvate is converted to lactate by lactate dehydrogenase (LDH). This causes acidification and excess lactate is exported from the cell. Pentose phosphate pathway (left section). G6P is converted to 6-phospho-gluconolactone by G6P dehydrogenase (G6PDH), relying on production of G6P that is increased by PKB/Akt signalling. This reaction produces NADPH, required to reduce oxidised glutathione (GSSG) to GSH. GSH is a critical antioxidant in the cell, scavenging reactive oxygen species (ROS). 6-phospho-gluconolactone can be returned to the glycolytic pathway via further oxidative and non-oxidative reactions.

PKB/Akt substrates account for other metabolic effects of insulin and IGF1: increased glucose uptake and enhanced glycolytic rate (Figure 2). The increase in glucose uptake induced by insulin is effected by increased translocation of storage vesicles containing GLUT4 glucose transporters to the plasma membrane. This process is regulated by Rab GTPases, inactivation of which is enhanced by Rab GTPase-activating proteins (RabGAPs). PKB/Akt phosphorylates two RabGAPs, Tbc1d1 and Tbc1d4 (more generally known as AS160) [67,68]. This enhances GLUT4 vesicle trafficking, probably by reducing binding of Tbc1d1/4 to Rabs. Insulin promotes phosphorylation of AS160/Tbc1d4 in the heart, and this is involved in translocation of GLUT4 and the fatty acid translocase CD36 to the cell surface [69]. The increase in the rate of glycolysis induced by insulin is mostly related to phosphorylation of the cardiac isoform of 6-phosphofructo-2-kinase/fructose 2,6-bisphosphatase (also known as phosphofructokinase 2; PFKB2) downstream of PDK1 and probably a result of direct phosphorylation by PKB/Akt [70–72]. PFKB2 is a bifunctional enzyme with an N-terminal kinase domain and C terminal phosphatase domain that regulates both production and degradation of fructose 2,6-*bis*phosphate (Figure 2). Phosphorylation of the C-terminal phosphatase domain by PKB/Akt selectively inhibits this activity, but the kinase remains active with continued production of fructose 2,6-*bis*phosphate, an allosteric regulator that promotes glycolysis and inhibits gluconeogenesis by acting on phosphofructokinase 1. PKB/Akt also phosphorylates hexokinase II (HKII) [73], the key enzyme required in insulin-sensitive tissues for production of glucose 6-phosphate in the first step of the glycolytic and pentose phosphate pathways. The latter contributes to the antioxidant capacity of the cell, in addition to provision of nucleosides. However, HKII also operates at the mitochondria to inhibit apoptosis [74], an area of considerable research in the heart. Indeed, studies of HKII in the heart have generally focused on cardioprotection and its role in preventing opening of the mitochondrial permeability transition pore (mPTP) with consequent inhibition of apoptosis [75], rather than its role in metabolism.

#### Other PKB/Akt substrates associated with cell survival

Apart from effects on metabolism, PKB/Akt phosphorylates a range of substrates with direct and/or indirect effects on cardiac cell survival (Figure 1C).

Endothelial nitric oxide synthase (eNOS) is phosphorylated by PKB/Akt on Ser1177 [76–78]. This residue is generally unphosphorylated in unstimulated cells and phosphorylation of endogenous enzyme increases production of NO [77]. This increased production of NO is associated with vasodilation of the blood vessels, reducing blood pressure and workload on the heart. It also reduces blood vessel reactivity, suppressing inflammation and reducing the potential for platelet activation [79]. Overall, this is expected to enhance cardiomyocyte survival. However, eNOS regulation is complex with an additional inhibitory phosphorylation site (Thr495) that may be constitutive in resting cells, and dephosphorylation of this site is required for activation. Moreover, the enzyme can become uncoupled and, instead of producing NO, eNOS can switch to production of potentially damaging O_2_^● −^.

PKB/Akt phosphorylates two proteins in the pathway for interferon production. Here, activation of cGAS (cyclic GMP AMP synthase) by viral infection or DNA from damaged mitochondria leads to production of the second messenger cGAMP (cyclic guanosine monophosphate-adenosine monophosphate) [80,81]. cGAMP activates STING (stimulator of interferon genes) which signals via the protein kinase TBK1 (TANK binding kinase 1) to phosphorylate and activate IRF3 (interferon regulatory factor 3), a key transcription factor required for interferon production. cGAS is phosphorylated on Ser291 by PKB/Akt, reducing its activity and production of cGAMP [80]. PKB/Akt also phosphorylates and inhibits TBK1 on Ser172 [82]. The net effect of PKB/Akt is to reduce interferon production, potentially enhancing cell survival.

Other direct PKB/Akt substrates include Forkhead transcription factors of the FoxO family, phosphorylation of which by PKB/Akt results in their retention in the cytoplasm, reducing expression of pro-apoptotic genes [83,84]. Forkhead transcription factor phosphorylation is increased by insulin or IGF1 in cardiomyocytes and the heart, downstream of PKB/Akt [85] and, although this is associated with a reduction in cardiomyocyte death, FoxO transcription factors also affect cardiac metabolism potentially influencing glucose utilisation [86,87]. Phosphorylation of Forkhead transcription factors appears to be mediated by the dually-phosphorylated form of PKB/Akt(Thr308/Ser473) with a requirement for Ser473 phosphorylation to extend the PKB/Akt substrate profile [59].

#### PKB/Akt signalling via mTORC1

Other effects of PKB/Akt are mediated by mTORC1, particularly in relation to the regulation of protein synthesis and ribosome biogenesis (Figure 1C) [88–90]. mTORC1 acts in several ways to enhance mRNA translation. A key mediator is p70 ribosomal S6 kinase (p70S6K) which is phosphorylated and activated downstream of mTORC1 in cardiac systems, as in other tissues [91]. p70S6K not only phosphorylates the small ribosomal subunit RPS6 to increase translation but also enhances recruitment of mRNAs with a 5′ terminal oligopyrimidine (TOP) sequence to polyribosomes for efficient translation. In cardiomyocytes, 5′-TOP mRNAs regulated by insulin are primarily those encoding the ribosomal subunits [92], so the net effect is to increase the translational machinery. Complementary effects of mTOR are to increase ribosomal RNA synthesis and processing [89]. p70S6K also influences the rate of initiation of translation of mRNAs by phosphorylating a number of eukaryotic initiation factors [90]. A second important group of substrates is the 4E-binding proteins (4E-BPs) which bind to eukaryotic initiation factor 4E (eIF4E). eIF4E interacts directly with the 7-methyl-GTP cap of mRNAs and eIF4E availability is a limiting factor. Phosphorylation of 4E-BPs in cardiomyocytes releases eIF4E, increasing availability of eIF4E for binding to the cap structure [60]. mTORC1 also increases the rate of protein synthesis by enhancing the rate of elongation [91]. Thus, mTORC1 signalling increases both the capacity and rate of protein synthesis in the heart. mTORC1 has a secondary effect to decrease autophagy, inhibiting ULK1 and preventing autophagic destruction of organelles [93]. This may account for the focused mechanism by which insulin inhibits protein degradation in the heart [20]. A final consideration for mTORC1 is that it increases expression of HKII [73] which (as discussed above) may have a dual effect on metabolism, increasing production of glucose 6-phosphate for use in glycolysis or the pentose phosphate pathway (Figure 2).

### Activation of the ERK1/2 cascade

Prior to the identification of PKB/Akt, microtubule-associated protein 2 [MAP2-kinase, later renamed mitogen-activated protein kinase (MAPK)] was identified by the Sturgill group as being associated with insulin signalling in 3T3-L1 adipocytes [94]. This particular 42 kDa protein became known as ERK2, and the related 44 kDa isoform as ERK1. INSRs and IGF1Rs activate the ERK1/2 cascade via guanine nucleotide exchange factors for the small G protein Ras (RasGEFs), including SOS1 and 2 that are recruited to the receptor complex by adapter proteins (e.g. Grb2 [95]) (Figure 1B). RasGEFs promote GTP-loading of Ras, which initiates signalling via RAF kinases. RAF kinases become activated and phosphorylate/activate MAPK kinases 1/2 (MKK1/2) which phosphorylate and activate ERK1/2 [96]. In contrast with their dominant effects on PKB/Akt, insulin and IGF1 have a minor effect on ERK1/2 activation in cardiomyocytes compared with other growth factors or hypertrophic agonists acting through G-protein-coupled receptors (GPCRs) [61]. It is not clear why insulin has a more pronounced effect on ERK1/2 activation in other systems, but it may relate to differentiation status, tissue-specificity or spatial organisation of intracellular signalling components as debated previously [97].

## INSRRs: sensing alkaline pH

### INSRR structure

INSRRs were identified soon after INSRs and IGF1Rs [9], but remained ‘orphan’ receptors for many years, having no known ligand. INSRRs have high homology with INSRs and IGF1Rs in the tyrosine kinase domain, but the extracellular α-subunit is divergent and does not bind insulin or IGFs (Figure 1A). The molecular phylogeny of the three insulin receptor family members indicates that whereas INSRs and IGF1Rs are expressed in all vertebrates, INSRRs are not found in teleost fish [98]. In addition, exon 11 is conserved between INSRRs and INSR-B receptors, but the alternatively spliced INSR-A receptor (that binds IGF2) appears to be exclusively expressed in mammals. The C-terminus of INSRRs is truncated compared with INSRs and IGF1Rs, producing a smaller β-subunit but, with this exception, the high homology in the intracellular region means that phospho-specific antibodies designed for conserved sites in the β-subunit recognise all three receptors and can be used to assess activation of INSRRs [62]. INSRR mRNA expression is highest in kidney, leading to a supposition that it was a kidney-specific receptor, but it is found at lower levels in other tissues including heart (∼20% that of kidney), pancreas and brain [99–101]. The receptors themselves are expressed in specific cells and subdomains, and (for example) INSRRs are found at the basolateral surface of intercalated B cells in kidney [102]. With such tight localisation, overall tissue or cellular expression levels are not necessarily reflective of functional significance. Like IGF1Rs, INSRRs form hybrid receptors with INSRs and these hybrids are not transactivated by insulin [103,104].

### Alkali-sensing role of INSRRs

Deyev et al. [105] established that INSRRs operate as alkali sensors: exposure to extracellular alkaline pH_o_ (pH_o_>8.0) causes a conformational change in the α subunit that activates the intracellular tyrosine kinase resulting in Tyr-phosphorylation of the β-subunit with activation of downstream signalling. INSRRs are important in maintaining acid-base balance. Thus, alkalosis in mice *in vivo* activates INSRRs in the kidney and, although INSRR null mice have no overt phenotypic abnormalities [106], they have an impaired renal response to HCO3^−^ loading resulting in metabolic alkalosis [105]. The focus on INSRRs has been their function in kidney, but the fact that alkaline pH_o_ increases cardiac protein synthesis to the same degree as insulin [24] suggests that INSRRs are important in the heart. INSRRs are expressed in the heart, enriched in membranes associated with T-tubules (invaginations in the sarcolemma designed to maximise efficiency of ion-exchange during the cardiac cycle), and are activated in rat hearts perfused at alkaline pH_o_ [62]. As with IGF1Rs, INSRRs appear to operate in the heart as hybrid receptors with INSRs and/or IGF1Rs (these cannot be easily distinguished, having similar molecular weight), and alkaline pH_o_ causes Tyr-phosphorylation of both INSRRs and INSRs/IGF1Rs. On the basis of the relative expression levels of the three insulin receptor family members and, with 90% of IGF1Rs forming hybrid receptors with INSRs, it is predicted that virtually all of the INSRRs should be present in cardiomyocytes as INSR/INSRR hybrids. Overall, it is highly probable that INSRRs expressed in T-tubules in the heart respond to alkaline pH_o_.

### Activation of protein kinase signalling by INSRRs

As might be expected, given the homology between INSRRs and INSRs/IGF1Rs, INSRRs have similar effects on intracellular signalling and, in kidney, there is Tyr-phosphorylation of IRS1 with activation of PKB/Akt [105]. In the heart, perfusion at alkaline pH_o_ results in phosphorylation of PKB/Akt on Thr308 and Ser473 (indicative of activation of mTORC1 and mTORC2), with associated phosphorylation of GSK3α/β, p70S6K and RPS6 [62]. The degree of activation is similar to that induced by insulin and presumably accounts for the effects of alkaline pH_o_ on cardiac protein synthesis [24]. The mechanism for triggering the PKB/Akt signalling pathway is not resolved, since INSRRs lack the direct binding sites for PI3Ks that are found at the C-terminus of INSRs/IGF1Rs [39]. It is also unclear if INSRRs recruit IRS1/2 directly. IRS1/2 bind to INSRs and IGF1Rs close to the transmembrane domain and, although the sequence for IRS1/2 binding is conserved in INSRRs, pull-down experiments failed to identify IRS1/2 binding to the peptide sequence of these receptors [39]. The most likely mechanism is via INSRs/IGF1Rs since INSRRs are almost certainly all expressed as hybrid receptors, and the conformational change induced in the INSRR hemi-receptor is potentially sufficient to trigger activation and transphosphorylation of its INSR/IGF1R partner. In contrast to insulin, perfusion of hearts at alkaline pH_o_ activates ERK1/2 to a significant degree [62]. This could be a direct INSRR-mediated effect, possibly mediated via the adapter protein Shc which has been detected in the INSRR interactome and can facilitate activation of Ras [39,107]. The site for interaction of Shc with INSRs/IGF1Rs is the same as for IRS1/2, so lack of binding of IRS1/2 may be permissive for Shc binding [39]. However, other stress-responsive MAPKs (JNKs and p38-MAPKs) are activated in hearts perfused at alkaline pH_o_ [62], so activation of all the MAPKs may be receptor-independent and simply reflect a degree of cellular stress.

### Functional role of INSRRs in the heart

**Figure 3:**
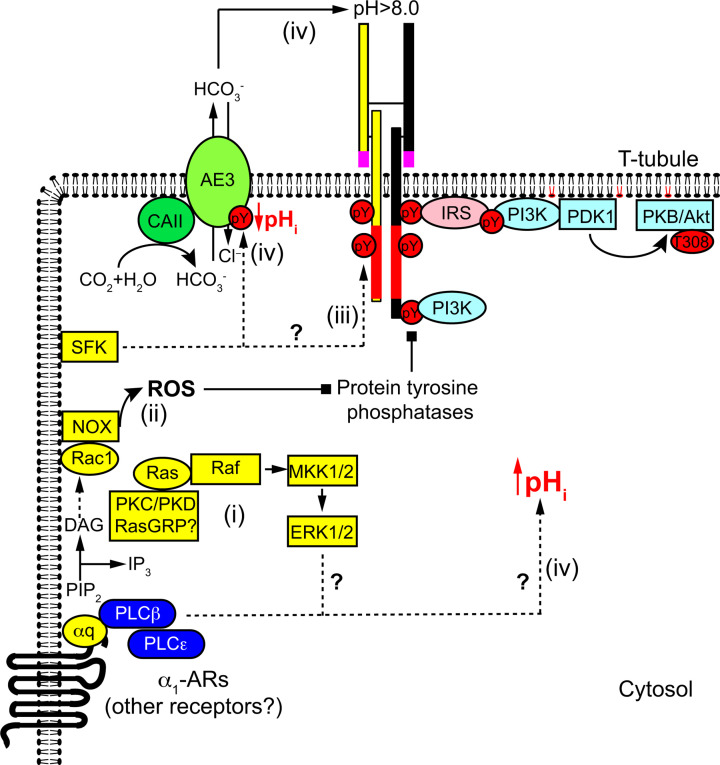
Cross-talk from α_1_-adrenergic receptors (α_1_-ARs) to INSR/INSRR receptors in the heart: possible mechanisms (i) α_1_-ARs are G-protein-coupled receptors that couple through Gαq to activate phospholipase C (PLC) β and possibly PLCε. PLCβ/ε hydrolyses phosphatidylinositol 4,5 *bis*phosphate (PIP_2_) to produce diacylglycerol (DAG) which activates protein kinase C (PKC) family members and guanine nucleotide exchange factors for Ras (e.g. RasGRP). This results in activation of the ERK1/2 cascade that affects downstream signalling and gene expression. (ii) Activation of the small G protein Rac1 results in an increase in production of reactive oxygen species (ROS) via NADPH oxidases (NOX). ROS inhibit protein tyrosine phosphatases (PTPs), and this may lead to increased tyrosine phosphorylation of INSRs/INSRRs, with receptor activation and downstream signalling. (iii) Src family kinases (SFKs) may be activated, possibly by phosphatidic acid produced by phospholipase D (activated by PKC; pathway not shown). SFKs may phosphorylate INSRs/INSRRs directly to cause receptor activation. (iv) α_1_-ARs increase intracellular pH_i_ via an unknown mechanism and this needs to be neutralised. SFKs phosphorylate anion exchanger 3 (AE3) to increase exchange of Cl^−^ and HCO_3_^−^ delivering HCO_3_^−^ to the extracellular space. Intracellular HCO_3_^−^ is probably generated by carbonic anhydrase II (CAII). Simultaneous production of H^+^ reduces intracellular pH_i_. Extracellular HCO_3_^−^ may increase extracellular pH_o_ to activate INSRRs via a conformational change of the extracellular α-subunit.

INSRRs have an obvious role in kidney as alkali sensors, but the potential role in heart is more obscure given that INSRRs are only activated when exposed to pH_o_>8.0 and blood pH as high as 7.6 is associated with mortality [108]. However, cardiac contraction is regulated by rapid ion fluxes throughout the cardiac cycle and it is essential that ion-balances, including intracellular and extracellular pH, are managed and normalised. INSRRs appear to be concentrated in transverse T tubule membranes [62]. The purpose of T tubules is to facilitate rapid contraction/relaxation, but the small volumes within these invaginations result in ion microdomains [109,110] that may generate a sufficiently high pH_o_ to activate INSRRs (Figure 3). Alternatively (or additionally), INSRRs (like INSRs or IGF1Rs) may be activated intracellularly. One mechanism may involve oxidative stress which can be generated intracellularly in microdomains by, for example, NADPH oxidases [111]. Oxidative stresses inhibit tyrosine phosphatases, with a net effect to increase Tyr-phosphorylation and activation of insulin family receptors, as occurs with the ‘insulinomimetic’ combination of H_2_O_2_ and/or vanadate treatment [112]. Furthermore, there could also be tyrosine phosphorylation of the β subunit by non-receptor tyrosine kinases (e.g. Src family kinases), potentially a consequence of cross-talk from other receptors resulting in transactivation of insulin family receptors [113].

## Cross-talk from α_1_-adrenergic receptors to insulin family receptors in the heart

As anabolic hormones, insulin and IGF1 promote cardiac growth, playing a major role in coupling nutritional status to cell size rather than necessarily contributing to cardiac hypertrophy. The most potent hypertrophic agonists for the adult heart (e.g. endothelin-1, α_1_-AR agonists) act through Gq protein-coupled receptors (GqPCRs) [114]. RTKs and GPCRs are generally considered to activate discrete signalling pathways, with RTKs initiating the signal via tyrosine phosphorylation (as described above for the insulin receptor family) whilst GPCRs signal through heterotrimeric G proteins that initiate downstream events via second messenger systems: Gq signalling induces hydrolysis of PIP_2_ to produce diacylglycerol and inositol 3,4,5 *tris*phosphate (Figure 3).

In many systems, there is cross-talk from GPCRs to RTKs, and downstream signalling emanates from RTKs [113]. In the heart, it is particularly well-recognised that epidermal growth factor receptors may be transactivated by different GPCRs [115]. There are also reports of GPCR transactivation of INSRs and IGF1Rs in proliferating cell lines with, for example, activation of INSRs by bradykinin or angiotensin II in INSR-overexpressing cells, possibly acting in a multireceptor complex and/or in association with β-arrestins [116,117], and transactivation of IGF1Rs by endothelin 1 in prostate cancer cells [118]. Recent data extend the concept to the heart, and activation of PKB/Akt by α_1_-AR agonists in perfused hearts or cardiomyocytes is inhibited by linsitinib [62], a highly selective inhibitor of the insulin receptor family tyrosine kinases [119]. Activation of ERK1/2 is also partially suppressed by linsitinib, suggesting that some of the signal is also mediated via one or more of the insulin family receptors. It appears that transactivation of insulin family receptors by α_1_-AR agonists is functionally important since linsitinib inhibits the increase in cell size induced in cultured cardiomyocytes by α_1_-AR agonists [62]. Surprisingly, it enhances cardiac hypertrophy induced by α_1_-AR stimulation *in vivo* even in the short term, but this is associated with increased interstitial fibrosis indicative of a switch towards maladaptive hypertrophy predicted to predispose to heart failure. It could be argued that insulin family receptor signalling contributes independently to cardiac hypertrophy induced by α_1_-ARs. However, linsitinib alone has no baseline effect on cardiac function or dimensions, at least over the short term.

The mechanism for activation of insulin family receptors by α_1_-AR agonists remains to be clarified, but there are several possible mechanisms (Figure 3). As mentioned in the previous section in relation to INSRRs, one or more of the receptors may be activated as a consequence of intracellular signalling (rather than extracellular stimulation) that increase Tyr-phosphorylation and activation of the β-subunit. This may result from direct phosphorylation by other tyrosine kinases (e.g. Src family kinases [120]) or could be a consequence of oxidative stress whether resulting from increased flux through the mitochondrial electron transport chain as a consequence of increased contractility or activation of NADPH oxidases [121]. However, INSRRs may be functionally relevant as alkali sensors in the heart, being triggered by increased pH_o_ in the T-tubules, in which case it is necessary to consider the cellular physiology relating to α_1_-AR stimulation. α_1_-AR agonists increase cardiomyocyte contractility. This is, at least in part, a result of an increase in pH_i_ of the order of 0.1–0.2 pH unit, which increases myofilament sensitivity to Ca^2+^, producing an increase in the force of contraction and rate of relaxation [122–125]. This increase in intracellular pH_i_ requires normalisation and one of the main systems involves anion exchanger 3 (AE3), a Cl^−^/HCO_3_^−^ exchanger that extrudes HCO_3_^−^ from the cell, with carbonic anhydrases providing a source of HCO_3_^−^ [126,127]. AE3 is expressed in the T tubules in cardiomyocytes [128], and therefore has potential to increase pH_o_ in close proximity to INSRRs. Interestingly, cardiomyocytes from AE3-/- knockout mice, though smaller than those of wild-type littermates, do not hypertrophy in response to α_1_-AR stimulation with no increase in size, protein synthesis or hypertrophic gene markers [129]. Inhibitors of carbonic anhydrases also abolish α_1_-AR agonist-induced cardiomyocyte hypertrophy and the response is similarly ablated in cardiomyocytes from mice lacking an intracellular isoform of carbonic anhydrase, CAII [130,131]. There is, therefore, potential for INSRRs to become activated as a consequence of increased pH_o_ in T tubules.

Further research is clearly necessary to address a number of different questions that arise. With respect to α_1_-ARs, it is important to determine which of the insulin family receptors are transactivated in response to α_1_-AR stimulation, and if INSRRs are (as we predict) involved in the response *in vivo*. It is also important to establish if transactivation occurs intracellularly or via an extracellular system. It would be interesting to explore whether other GPCR agonists also transactivate insulin family receptors since, as mentioned above, endothelin-1 and bradykinin can activate INSRs or IGF1Rs in other cells [116–118], and both have potent effects on cardiomyocyte signalling [132–134]. Apart from situations of receptor cross-talk, it is also worth considering how pathological stresses may influence insulin family receptor signalling. For example, pressure overload enhances insulin receptor signalling [135], and ischaemia/reperfusion (as occurs in myocardial infarction) is associated with activation of PKB/Akt [136]. In the latter case, acidosis that develops on ischaemia is rapidly normalised on reperfusion, in association with various changes in ion fluxes, and elevated oxidative stress [137]. This would be expected to inactivate tyrosine phosphatases, potentially resulting in activation of insulin receptor family members.

### Insulin family receptor signalling and cardioprotection: when and how?

Insulin and IGF1 can be cardioprotective: insulin/IGF1 reduce infarct size in *ex vivo* models of myocardial infarction [138–143], and cardiomyocyte-specific expression of IGF1 in mice protects against myocardial infarction *in vivo* [144]. PKB/Akt signalling is particularly associated with cardioprotection [145,146]. Thus, insulin/IGF1 signalling via PKB/Akt promotes cell survival in the heart. However, hyperinsulinemia is not beneficial for the heart, being associated with diabetic cardiomyopathy and obesity-induced heart failure [147]. Moreover, not all studies are fully supportive of the concept that IGF1 is really cardioprotective [148]. Some of the discrepancies may reflect the increased reliance on genetically altered mice to assess the cardioprotective effects or otherwise of IGF1 or the different signalling intermediates and, even if a conditional and inducible system is used for gene manipulation, this results in changes of prolonged duration and abnormal stoichiometry that are not necessarily representative of the endogenous system. It is important to consider not just how insulin receptor family signalling is cardioprotective, but when it may be useful.

#### When?

Almost all studies of cardioprotection with insulin and IGF1 have used models of myocardial infarction with cardiomyocytes or the heart subjected to hypoxia/ischaemia followed by reoxygenation/reperfusion. In the ischaemic phase, cardiomyocyte energy production via oxidative phosphorylation in the mitochondria is compromised reducing contractile ability, the cytosol becomes acidified and there is a low level of oxidative stress. Injury is exacerbated on reoxygenation/reperfusion, due in part to a substantial increase in reactive oxygen species [149]. Cytosolic pH recovers, but this is associated with further damage [150]. Cardiomyocytes in the ischaemic zone die via necrosis, but additional apoptotic cell death continues in the peri-infarct zone and even in the remote myocardium, contributing to cardiac dysfunction. Apoptotic cell death occurs largely through the mitochondrial death pathway with opening of the mPTP, triggering activation of the caspase 9 → caspase 3 death cascade [151]. This is an acute stress that requires an initial acute treatment. Points for intervention include maintenance of cardiac energetics, prevention of mitochondrial dysfunction and mitochondrial-induced cell death, reduction of inflammation and reduction of oxidative stress, all of which may be influenced by insulin receptor family signalling in the short term. Another scenario in which the insulin signalling pathway may be beneficial is myocarditis in which activation of PKB/Akt may reduce the degree of inflammation and prevent cardiomyocyte dysfunction or loss. Whether or not the benefits of insulin receptor family signalling may be extrapolated to chronic heart diseases is likely to depend on the cardioprotective mechanism of action of insulin receptor family signalling and the underlying mechanism of disease. Here, a consideration may be that prolonged activation of insulin receptor signalling may be associated with desensitisation of the pathway.

#### Mechanisms of cardioprotection?

**Figure 4:**
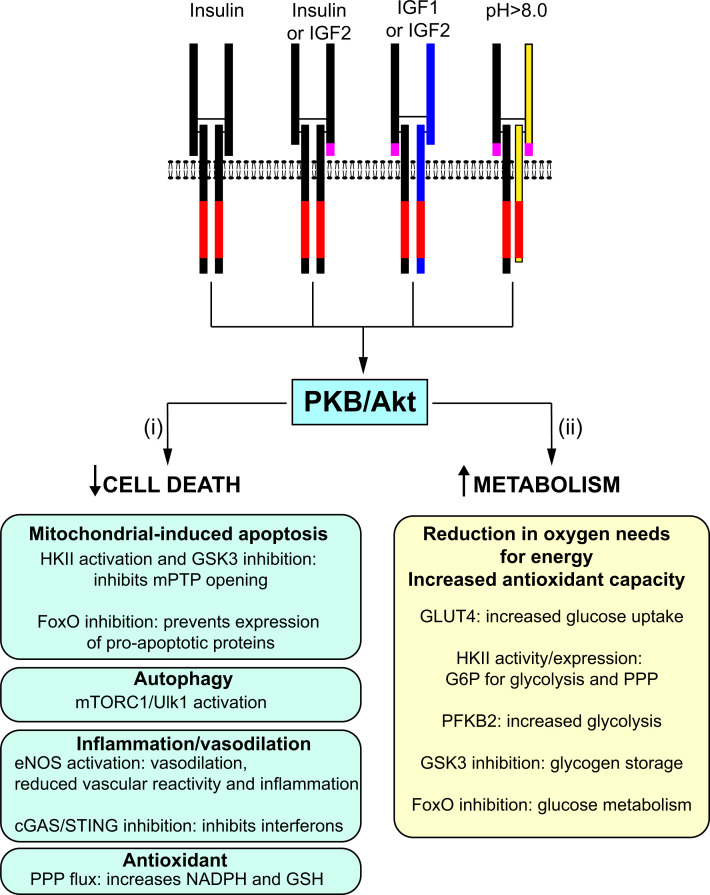
Possible mechanisms of cardioprotection resulting from stimulation of insulin receptor family members Activation of insulin receptor family members results in activation of protein kinase B (PKB, also known as Akt). (i) PKB/Akt signalling influences cell death by reducing mitochondrial-dependent apoptosis, increasing hexokinase II (HKII) and inhibiting glycogen synthase kinase 3 (GSK3) activities. This prevents opening of the mitochondrial permeability transition pore (mPTP). Inhibition of FoxO transcription factors reduces expression of pro-apoptotic proteins. Signalling via mTORC1 inhibits autophagy via Ulk1. Activation of eNOS may reduce cell death, acting to promote vasodilation and, thus, reduce workload. It also reduces vascular inflammation. Phosphorylation of cGAS and STING inhibits interferon production. Increased flux through the pentose phosphate pathway (PPP) increases antioxidant capacity in the cell by increasing production of NADPH and glutathione (GSH). (ii) PKB/Akt increases metabolism, providing the cell with options for reducing oxygen needs whilst sustaining energy production, and maintaining antioxidant capability via the NADPH/glutathione system. Glucose uptake is increased by translocation of GLUT4 to the membrane. HKII increases availability of glucose 6-phosphate (G6P) for both glycolysis and the pentose phosphate pathway (PPP). Phosphorylation of the cardiac isoform of phosphofructokinase 2 (PFKB2) inhibits the phosphatase activity, increasing production of fructose 2,6-*bis*phosphate, an allosteric activator of glycolysis. Glycogen storage is increased by GSK3 inhibition, providing a source of glucose during ischaemia. FoxO inhibition increases glucose metabolism.

PKB/Akt signalling may directly inhibit mitochondrial-induced cell death of cardiomyocytes and endothelial cells to maintain contraction and cardiac perfusion (Figure 4). As discussed above, direct phosphorylation of FoxO transcription factors by PKB/Akt (Figure 1C) is proposed to reduce synthesis of pro-apoptotic proteins [83–87]. This may provide longer term cardioprotection, but seems unlikely to account for acute protection afforded by insulin in models of myocardial infarction. A second system involves mitochondrial HKII that not only has a role in metabolism but also interacts with the mPTP and prevents channel opening [73]. Phosphorylation of HKII at the mitochondria by PKB/Akt may reduce the potential for apoptosis. In addition, mTORC1 increases HKII expression but, as with FoxO transcription factors, this seems unlikely to confer acute cardioprotection. mTORC1 can prevent autophagy and preserve organelles such as mitochondria in the cell [93]. Theoretically, this could be cardioprotective because potential energy production is preserved. However, autophagy is not necessarily detrimental and may even be beneficial because defective organelles (e.g. damaged mitochondria that may generate excessive reactive oxygen species) are cleared. Indeed, enhancement of autophagic processes is associated with increased longevity [152]. Possibly the strongest evidence for a direct effect of insulin receptor family signalling in preventing cell death relates to GSK3α/β which, as discussed above, is phosphorylated and inhibited by PKB/Akt [63]. GSK3α/β are associated with the mitochondria and GSK3α/β activity increases the probability of mPTP opening and promotes cardiomyocyte apoptosis, so some of the protective effects of PKB/Akt may be via inhibition of GSK3-mediated apoptosis [153].

There is, therefore, evidence that insulin/IGFs may prevent cell death directly, but insulin and IGFs have profound effects on metabolism (Figure 2), and this may account for a large part of the cardioprotection they afford (Figure 4). Indeed, this was the basis for some of the earliest studies of the cardioprotective effects of insulin in the 1960s/1970s, demonstrating that glucose-insulin-potassium (GIK) treatment protects the myocardium from ischaemic damage in a dog model of myocardial infarction [154]. Under oxygenated conditions, cardiac energy production derives largely from fatty acid metabolism and oxidative phosphorylation, with heavy reliance on availability of oxygen. The hypothesis was that reducing oxygen consumption by the heart should limit ischaemic damage resulting from coronary artery occlusion and one way to achieve this is to enhance glycolysis using insulin with provision of additional glucose [155]. Increased glycolysis could potentially compensate for the reduction in mitochondrial ATP production resulting from even 50% oxygenation [156]. We now have some insight into the mechanism of action (PKB/Akt phosphorylation of RabGAPs promotes GLUT4 translocation to the membrane; phosphorylation of PFKB2 increases the rate of glycolysis (Figure 2)), but insulin was already known to increase glucose uptake and glycolytic flux, observations that continue to be upheld [157]. Whilst increasing glucose availability and glycolytic flux may not salvage cardiomyocytes in the full ischaemic zone, it may be sufficient to preserve cardiomyocytes in border zones for damage limitation.

HKII and GSK3α/β are generally considered to exert their cardioprotective effects by preventing opening of the mPTP and suppressing apoptosis (Figure 4). However, they are key metabolism enzymes. HKII directs glucose into the glycolytic pathway (Figure 2), associating with the mPTP at the mitochondria where the enzyme has ready access to mitochondrial-generated ATP for production of glucose 6-phosphate [158]. It then allows the ADP that is produced to pass straight back into the mitochondrion for oxidative phosphorylation [159]. Glucose 6-phosphate is not just required for glycolysis but is also the first intermediate of the pentose phosphate pathway which produces NADPH required to maintain reduced glutathione levels, a vital component in the antioxidant repertoire of the cell [160]. This pathway is strictly regulated, with the next step of the pathway being highly dependent on the ratio of NADPH:NADP^+^: as NADP^+^ rises (as a result of increased oxidative stress in the cell), flow through the pentose phosphate pathway is rapidly increased [161]. Lack of substrate compromises this response, resulting in enhanced oxidative stress and, potentially, causing activation of the mitochondrial death pathway via mPTP opening.

GSK3α/β phosphorylates and inhibits glycogen synthase (Figure 1C), a metabolic effect that should not be ignored, and PKB/Akt phosphorylation/inhibition of GSK3α/β has a net effect to increase glycogen levels in the cell [162]. The protective benefit of this is not immediately obvious, but glycogen is an important energy source, being mobilised in ischaemia as a result of increased phosphorylase kinase and glycogen phosphorylase activities [163]. This probably occurs as cytosolic Ca^2+^ concentrations increase and Ca^2+^ binding to the inhibitory δ subunit of phosphorylase kinase causes it to be released from the complex, increasing enzyme activity [164,165]. Phosphorylase kinase phosphorylates and activates glycogen phosphorylase which hydrolyses glycogen to produce glucose 1-phosphate. Glycogen stores provide a glycolytic reserve and potentially protect against cardiac damage in anoxia [166], so at least some of the protective effects associated with loss/inhibition of GSK3 activity, particularly in genetically altered mice, may be associated with increased glycogen stores, as in other models associated with increased glycogen accumulation (e.g. overexpression of AMPKγ [167]).

Insulin receptor family signalling clearly has acute effects on metabolism to provide cardioprotection. However, there are longer term effects on gene expression that also impact on metabolism. First, mTORC1-dependent up-regulation of HKII increases glucose utilisation for both glycolysis and to support antioxidant capacity of the cell via the pentose phosphate pathway [73]. In addition, whilst FoxO transcription factors are recognised as drivers of expression of pro-apoptotic genes, they also to have a significant impact on metabolism, and inhibition of FoxO may support a switch from fatty acid metabolism to glucose utilisation [168]. Such changes are likely to influence cardioprotection in the longer term, potentially during remodelling following myocardial infarction or, possibly, in other cardiac diseases.

Phosphorylation and activation of eNOS by PKB/Akt is often viewed as being cardioprotective (see, for example, [79]). eNOS is expressed at highest levels in endothelial cells, and increased production of NO in blood vessels to promote vasodilation is clearly potentially beneficial because of the reduced workload on the heart (Figure 4). However, in the context of high levels of oxidative stress, NO may combine with O_2_^● −^ to produce the peroxynitrite anion (ONOO^● −^), a highly reactive species associated with nitrosative stress that can cause cell death [169]. Furthermore, if eNOS becomes uncoupled, it may generate O_2_^● −^ rather than NO, enhancing oxidative stress. Although eNOS is highly expressed in endothelial cells, it is also expressed in cardiomyocytes, and NO produced by either cell type in the heart may have autocrine/paracrine effects on cardiac function [170]. These effects may be beneficial. For example, most cardiac perfusion occurs during diastole and NO may reduce diastolic stiffening to improve cardiac filling and, therefore, perfusion [171]. However, the data are not always consistent and, as in blood vessels, the effects may depend on the context, particularly with respect to the degree of oxidative stress (which will be high following myocardial infarction) and disease state [170].

#### Therapeutic strategy for cardioprotection?

It is over 100 years since insulin was discovered and 40 years since the receptor structure was first investigated. The black box from 1979 now contains many signalling intermediates [172] and, though much of what was known about insulin effects in the 1970s is what we know now, we now have an idea of how it happens, even if much still remains to be learned. Irrespective of precise mechanism of action, there are many potential points of intervention in the black box which became the PKB/Akt signalling pathway that may be targeted for cardioprotection. Strategically, targeting an extracellular domain of a receptor is generally an easier option than developing small molecule inhibitors of sufficient specificity or selectivity for intracellular action. One problem for the insulin family receptors has been the pleiotropic effects of insulin and IGF1 throughout the body, so activating INSRs/IGF1Rs to protect the heart is potentially undesirable unless in the very short term. INSRR expression is more restricted and the recent understanding of how their conformation is regulated by pH [173–176] will almost certainly provide insight into how they may best be activated. Developing synthetic agonists to target insulin receptor family members is not an easy option, and even a substitute for insulin has proved difficult to develop. However, as in cancer, one therapeutic option might be an activating monoclonal antibody to target INSRRs at least one of which has previously been produced [50]. Peptide mimetics or aptamers may prove alternative viable options.

## Summary and perspectives

Insulin and IGF1 are anabolic hormones and writing this overview enabled a rediscovery of their metabolic effects. As we describe above, many aspects of PKB/Akt signalling impinge on cardiac metabolism and, in that context, it is not unreasonable that the major conclusion is that cardioprotection afforded by insulin and IGF1 is largely due to regulation of metabolism. Key effects are the increases in glucose uptake and glycolytic flux, and increasing the rate of glycolysis was suggested as a therapeutic option for improving cardiac function during cardiac anoxia as early as 1975 [156]. Interestingly, given that we now know that INSRRs are alkali sensors, another suggestion from Scheuer in the mid-1970s [156] was that ‘the protection of the ischemic or hypoxic heart by alkalosis may be a feasible approach’, because of the effects on glycolysis [177]. The greatest problem with increasing glycolytic flux in hypoxic conditions as a therapeutic option is probably the associated accumulation of lactic acid and intracellular acidification, but both are normalised on reperfusion/reoxygenation, and any interim damage may be offset by cardiomyocyte preservation. There are clearly other effectors of insulin signalling that may reduce cardiomyocyte or endothelial cell death, thus preserving cardiac function (Figure 4). Here, a consideration is whether preservation of cells that are clearly dysfunctional (or cell death programmes would not have been initiated) is genuinely beneficial in the absence of other systems to salvage and repair the cell. Clearly, indirect effects to reduce cardiac workload by, for example, vasodilation resulting from NO production or reducing inflammation will be beneficial to a heart in jeopardy. Perhaps the combinatorial effects of coupling bona fide protective systems affecting cardiac cell apoptosis with metabolic effects is the reason that insulin receptor family signalling is so potently cardioprotective.

## Open Access

Open access for this article was enabled by the participation of University of Reading in an all-inclusive *Read & Publish* agreement with Portland Press and the Biochemical Society under a transformative agreement with JISC.

## Abbreviations

4E-BP: 4E-binding protein
AR: adrenergic receptor
CA: carbonic anhydrase
cGAMP: cyclic guanosine monophosphate-adenosine monophosphate
cGAS: cyclic GMP AMP synthase
eIF4E: eukaryotic initiation factor 4E
eNOS: endothelial nitric oxide synthase
ERK1/2: extracellular signal-regulated kinase 1/2
GAP: GTPase activating protein
GEF: guanine nucleotide exchange factor
GPCR: G-protein-coupled receptor
GSK: glycogen synthase kinase
HK: hexokinase
IGF: insulin-like growth factor
IGF1R: IGF1 receptor
IGFBP: IGF-binding protein
INSR: insulin receptor
INSRR: insulin receptor-related receptor
IRF3: interferon regulatory factor
IRS: insulin receptor substrate
M6PR: mannose 6 phosphate receptor
MAPK: mitogen-activated protein kinase
MKK: MAPK kinase
mPTP: mitochondrial permeability transition pore
mTOR: mammalian target of rapamycin
mTORC: mTOR complex
PDK1: phosphoinositide-dependent kinase 1
PI3K: phosphoinositide 3′ kinase
PKB: protein kinase B
RTK: receptor tyrosine kinase
STING: stimulator of interferon genes
TBK1: TANK binding kinase 1
